# Histaminergic interneurons in the ventral nerve cord: assessment of their value for Euarthropod phylogeny

**DOI:** 10.1186/s40851-019-0151-1

**Published:** 2019-12-23

**Authors:** Maite Maurer, Janina Hladik, Thomas M. Iliffe, Torben Stemme

**Affiliations:** 1 Institute of Neurobiology, University of Ulm, Albert-Einstein-Allee 11, 89081 Ulm, Germany; 2 Department of Marine Biology, Texas A&M University at Galveston, 200 Seawolf Parkway, Galveston, TX 77553 USA

**Keywords:** Ground pattern reconstruction, Serial homology, Biogenic amines, Inhibitory transmitter, Neurophylogeny, Scorpion, Pseudoscorpion, Remipede, VNC, Evolution

## Abstract

Despite numerous approaches to the resolution of euarthropod phylogeny, mainly based on modern sequence information and traditional external morphology, the resulting hypotheses are often contradictory and leave many questions about euarthropod evolution unanswered. The comparison of developmental and structural aspects of the nervous system has shown to be a valuable contribution to the assessment of current phylogenetic hypotheses. One promising approach for the generation of new character sets is the morphology of transmitter systems and the discovery of individually identifiable neurons, which allow phylogenetic comparisons on the single cell level. In this context, the serotonin transmitter system has been investigated to a considerable degree. Studies to date have yielded important stimuli to our understanding of euarthropod relationships and the evolution of their nervous systems. However, data on other transmitter systems remain fragmented, and their value with respect to phylogenetic questions remains speculative. The biogenic amine histamine is a promising transmitter; a substantial amount of data has been reported in the literature and the homology of some histaminergic neurons has been suggested. Here, we present a comprehensive review of histaminergic neurons in the ventral nerve cord of Euarthropoda. Using immunocytochemical labeling of histamine combined with confocal laser-scanning microscopy, we investigated the transmitter system in phylogenetically relevant taxa, such as Zygentoma, Remipedia, Diplopoda, and Arachnida. By reconstructing ground patterns, we evaluated the significance of this specific character set for euarthropod phylogeny. With this approach, we identified a set of neurons, which can be considered homologous within the respective major taxon. In conclusion, the histaminergic system contains useful information for our understanding of euarthropod phylogeny, supporting the proposed clades Tetraconata and Mandibulata. Furthermore, this character set has considerable potential to help resolve relationships within the major clades at a deeper level of taxonomy, due to the considerable variability in neurite morphology.

## Introduction

The investigation of the anatomy of invertebrate nervous systems, and of euarthropod (Arthropoda sensu stricto, strictly the extant subphyla Chelicerata, Myriapoda, Crustacea, and Hexapoda) nervous systems in particular, has undergone a renaissance and received increasing attention in recent decades (e.g., [1, 2]). Especially from the comparative aspect, combining structural descriptions of nervous systems from non-model organisms with evolutionary aspects has created a wealth of studies since the first comparative approaches by Holmgren and Hanström about 100 years ago (e.g., [3, 4]). One fact that has fueled this field in recent decades is the still unresolved debate about phylogenetic relationships, due to often contradicting hypotheses based on traditional external morphology on the one hand and modern sequence information analyses on the other.

Currently, a close relationship of Crustacea and Hexapoda (building the Tetraconata or Pancrustacea) and the Myriapoda being the sister group to the Tetraconata (a clade termed Mandibulata) is widely accepted (e.g., [5–8]). However, alternative views on their relationship have been postulated, including concepts such as Tracheata/Atelocerata (Myriapoda + Hexapoda) and Myriochelata/Paradoxopoda (Chelicerata + Myriapoda) (e.g., [9, 10])). In particular, the internal phylogeny of the major taxa is far from clear. To give an example, it has been proposed that the Crustacea form a non-monophyletic taxon and that several crustacean subgroups (e.g., Copepoda, Branchiopoda, Malacostraca, Cephalocarida, Remipedia or Xenocarida (Remipedia + Cephalocarida) are more closely related to the Hexapoda than to the remaining Crustacea (reviewed in [11]). Similarly, the internal phylogeny of Chelicerata and Myriapoda remains under debate [12–15].

The nervous system has been shown to contain numerous rather robust informative character sets for evaluating current evolutionary scenarios [16, 17]. These characters have been used in cladistic analyses, resulting in comparably well-resolved phylogenies [18, 19]. This research has motivated detailed neuromorphological descriptions for many non-model organisms in recent years, uncovering the great diversity in invertebrate, and especially euarthropod, nervous systems [2].

As a special character set, individually identifiable neurons have been classified as a promising tool for re-evaluating the phylogenetic relationships within the Euarthropoda. Based on specific criteria for the homology of individually identifiable neurons established by Kutsch and Breidbach [20], these neurons might be homologized intra- and interspecifically. Along these lines, neuromorphologists have largely focused on the serotonin transmitter system [21–28]. Serotonergic neurons show clear advantages, which allow their individual identification and, consequentially, can be used for the analysis of evolutionary processes in Euarthropoda. First, only few serotonergic neurons are distributed in the euarthropod ventral nerve cord, which are mostly found in a repetitive pattern within single ganglia, suggesting serial homology. Second, due to the low number of serotonergic neurons, the characteristic neurite morphology can be traced rather easily, providing the basis for the reconstruction of evolutionary transformation in this transmitter system. Meanwhile, an impressive number of investigated taxa has accumulated in the literature, and ground patterns for major euarthropod taxa have been reconstructed (summarized in [24, 28]).

However, other neurotransmitter systems have not received comparable attention for inferring homologies on the single-cell level, making interpretations concerning the serotonergic system difficult. Many transmitters, for example GABA, are distributed rather globally in the ganglia of the ventral nerve cord (e.g., [29–32]), hampering the individual identification of single cells. Only the combination of backfill techniques and immunocytochemical labeling of GABA, supplemented by electrophysiological experiments, has enabled the detection of individually identifiable inhibitory motoneurons, known as common inhibitors, which have also been considered as a phylogenetically relevant character set [30, 33]. Neuropeptides like FMRF-amides, or allatostatins, constitute large families of similar neuropeptides, distributed throughout the nervous system (e.g., [34–39]). Various neuropeptides belonging to such a family might be labeled by the same (polyclonal) antibody, thus jeopardizing homologization of neurons on single cell level [40, 41].

One promising system for single cell level comparisons are neurons containing the biogenic amine histamine. This transmitter has been investigated in the ventral nerve cord of various euarthropod taxa (Table 1) where only a few histaminergic neurons are distributed in a serial, repetitive pattern. Although homology of certain histaminergic neurons has been suggested, rigorous analysis of the existing data and ground pattern reconstruction has not been performed, but seems to be feasible and is highly desirable [41].

**Table 1:** Euarthropod species, in which histaminergic neurons have been investigated within the entire ventral cord

Chelicerata	Merostomata	Xiphosura	*Limulus polyphemus*	[42, 43]
	Arachnida	Scorpiones	*Euscorpius italicus*	this study
	Arachnida	Pseudoscorpiones	*Chelifer cancroides*	this study
	Arachnida	Araneae (Araneomorphae)	*Cupiennius salei*	[44, 45]
	Arachnida	Araneae (Araneomorphae)	*Tegenaria atrica*	[45]
	Arachnida	Araneae (Araneomorphae)	*Lycosa tarentula*	[45]
	Arachnida	Araneae (Mygalomorphae)	*Psalmopoeus cambridgei*	[45]
Myriapoda	Chilopoda	Lithobiomorpha	*Lithobius forficatus*	[41]
	Diplopoda	Glomerida	*Glomeris marginata*	this study
Crustacea	Maxillopoda	Cirripedia	*Balanus nubilus*	[46]
	Maxillopoda	Cirripedia	*Semibalanus cariosus*	[46]
	Branchiopoda	Anostraca	*Artemia salina*	[47]
	Branchiopoda	Notostraca	*Triops cancriformis*	[48]
	Branchiopoda	Onychura	*Daphnia pulex*	[49]
	Branchiopoda	Onychura	*Daphnia magna*	[49]
	Malacostraca	Decapoda (Astacidea)	*Pacifastacus leniusculus*	[50]
	Malacostraca	Decapoda (Astacidea)	*Homarus americanus*	[50]
	Malacostraca	Decapoda (Astacidea)	*Procambarus fallax f. virginalis*	[51]
Hexapoda	Zygentoma		*Thermobia domestica*	this study
	Orthoptera	Caelifera	*Schistocerca gregaria*	[52]
	Orthoptera	Caelifera	*Locusta migratoria*	[52]
	Orthoptera	Ensifera	*Gryllus bimaculatus*	[53, 54]
	Diptera	Brachycera	*Drosophila melanogaster*	[55]
	Diptera	Brachycera	*Calliphora vomitoria*	[55]

The biogenic amine histamine acts as a fast inhibitory neurotransmitter [56, 57] and is known to be the universal transmitter of euarthropod photoreceptors (e.g., [46, 58–61]). Additionally, histamine is a major transmitter of the mechanosensory sensilla in *Drosophila melanogaster* [62, 63] and of some mechanosensory receptors in the spider *Cupiennius salei* [64]. Aside from detailed descriptions of histamine in the insect brain (for review see [59]), the role of histaminergic neurons in ganglia of the euarthropod ventral nerve cord is incompletely understood. Some evidence that histamine is involved in modulation of the neuromuscular system [65], the activity of the somatogastric ganglion [66], or the control of heartbeat rate [67] has been gained from crustacean representatives. Histamine has been shown to influence the central auditory pathway in the prothoracic ganglion of the cricket [68]. Hörner et al. [53] suggested multiple neuroactive functions of histamine released from wide-field interneurons in the ventral nerve cord of crickets, possibly affecting neuronal and non-neuronal systems. Recently, it was demonstrated that a single pair of mesothoracic histaminergic neurons innervate a subset of antennal lobe glomeruli in *Manduca sexta* [69]. This sensory-motor circuit has been identified in several moth species, but not in other insects including locusts, flies and butterflies, where these neurons build a motor-to-mechanosensory circuit [70].

The present article sets out (a) to review the existing literature concerning the distribution of histamine-immunoreactive neurons in the ventral nerve cord of Euarthropoda, (b) to fill crucial gaps in our knowledge of the morphology of these neurons in certain taxa of each major group of the Euarthropoda, (c) to reconstruct ground patterns for the investigated groups based on serial homologous neurons, and (d) to discuss these ground patterns in a phylogenetic framework in order to evaluate the potential of individually identifiable histaminergic neurons for phylogenetic approaches.

To reach these goals, we investigated the histaminergic system in representatives of all four major euarthropod taxa. In hexapods, we selected an apterygote representative belonging to the Zygentoma, a basal clade of insects. Furthermore, we added the remipede *Xibalbanus tulumensis*. These crustaceans play a key role in tetraconate evolution, as growing evidence from molecular sequence analyses indicates Remipedia to be the closest relatives of Hexapoda (summarized in e.g., [71, 72]). In the more basal euarthropod taxa Myriapoda and Chelicerata, we investigated the first representative of Diplopoda, *Glomeris marginata*, and added two arachnid taxa (Scorpiones, Pseudoscorpiones), in order to reach a broader taxon sampling in these rather neglected clades. Using immunocytochemical labeling of histamine and synapsin, combined with confocal laser-scanning microscopy, we were able to reconstruct the ground patterns for these taxa and to undertake a comprehensive comparison to already existing data.

## Materials and methods

### Experimental animals

Adult specimens of the firebrat *Thermobia domestica* (Packard, 1873) were purchased from http://www.terraristikshop.net and maintained at approximately 30 °C in fauna boxes equipped with water reservoirs. Egg carton and screwed paper served as forage and hiding place. Additionally, animals were fed with wheat bran.

Representatives of the pseudoscorpion *Chelifer cancroides* (Linnaeus, 1758) were collected from an old hayloft near Rinteln (Lower Saxony, Germany). Animals gathered under a wooden board covered with hay. After collection, animals were kept in jars with screwed paper for transport to the laboratory where they were sacrificed as soon as possible.

Pill millipedes *Glomeris marginata* (Villers, 1789) were collected in forests near Hannover and Rinteln (Lower Saxony, Germany) under deadwood and in the leaf litter. Collected animals were kept in the laboratory in fauna boxes filled with soil, leaf litter, and deadwood in moist environment until use.

Scorpions represented by *Euscorpius italicus* (Herbst, 1800) were collected in the foothills of the Alps at altitudes of about 800 m above sea level just north of Bozen in northern Italy. The adult animals were kept solitary in fauna boxes at temperatures between 18 and 25 °C. The boxes were filled with approximately 5 cm of soil as substrate and terra cotta shards served as hiding places. Small shallow water dishes were provided and feeding was carried out every two weeks with crickets (*Gryllus bimaculatus*).

Specimens of the remipede *Xibalbanus tulumensis* [73], originally described as *Speleonectes tulumensis* [74], were collected in Cenote Crustacea on the Yucatán Peninsula, Mexico by appropriately trained and experienced cave divers. Remipedes are found only in marine waters at depths of > 18 m, approximately 300 m horizontally from the cave entrance. Specimens were collected individually in 50 ml Falcon tubes containing water from the same depth. Within 4–6 h after collection, specimens were directly processed, as detailed below.

In the following experiments, at least ten specimens were analyzed for each species, except for *Xibalbanus tulumensis*, where three individuals were utilized (due to the relative rarity of this species).

### Dissection and fixation

Remipedia were cut into three parts of approximately 1.5 cm length and pre-fixed in 4% N-(3-Dimethylaminopropyl)-N′-ethylcarbodiimide hydrochloride (EDAC) (Calbiochem, Merck, Darmstadt, Germany) dissolved in phosphate-buffered saline (PBS, 10 mM sodium phosphate, 150 mM NaCl, pH 7.4; chemicals obtained from Merck) for 4 h at 4 °C and then post-fixed in 4% paraformaldehyde (PFA) (Sigma Aldrich, St. Louis, MO) in PBS overnight at 4 °C (protocol modified from [57]). Specimens were washed three times for 1 h in PBS, shipped and stored in PBS containing 0.1% sodium azide until use. The remipede ventral nerve cord was dissected in ice cooled PBS using fine forceps.

All other animals were cold anesthetized and dissected in PBS using fine forceps. Dissected nervous tissue was pre-fixed in 4% EDAC dissolved in PBS for 4 h at 4 °C and then post-fixed in 4% PFA in PBS overnight at 4 °C. After three washing steps of at least 15 min in PBS at room temperature, the ventral nerve cords were freed of remaining surrounding tissue and further processed for immunocytochemistry as whole mounts or vibratome sections.

### Immunocytochemistry on vibratome sections

After fixation, the nerve cords were briefly covered with poly-D-lysine (Merck) to achieve better connection between tissue and embedding medium. The removal of the poly-D-lysine was followed by embedding the tissue in 7% low melting point agarose (Carl Roth, Karlsruhe, Germany) dissolved in distilled water at approximately 40 °C. The preparations were cooled to 4 °C and the trimmed blocks were cut into 50–75 μm thick sections by using a VT 1000 S Vibratome (Leica, Wetzlar, Germany). Nerve cords were permeabilized for 1 h in 0.3% saponin (Sigma Aldrich) in PBS containing 0.5% Triton X-100 (Merck) (PBS-TX 0.5%), washed three times for 15 min each in PBS-TX 0.5%, and afterwards incubated for at least 3 h in 5% normal goat serum (Vector Laboratories, Burlingame, CA) in PBS-TX 0.5% as blocking solution. In the following step, the primary antibodies rabbit-anti-histamine (ImmunoStar, Hudson, WI, Lot: 1532001; final concentration 1:500) and mouse-anti-synapsin (Developmental Studies Hybridoma Bank, University of Iowa, 3C11; 1:75) were applied overnight at room temperature in blocking solution containing 1% Triton X-100. After three washing steps for 15 min in PBS-TX 0.5%, the preparations were incubated for at least 3 h at room temperature in the secondary antibodies goat-anti-rabbit Cy3-conjugated (Jackson ImmunoResearch, Cambridgeshire, UK, Lot 132,481) and goat-anti-mouse Alexa Fluor 488-conjugated (Invitrogen, Carlsbad, CA, Lot 1,907,294), each diluted 1:250 in blocking solution containing 0.5% Triton X-100. Preparations were washed two times in PBS-TX 0.5% and once with PBS for 15 min each and finally mounted on adhesive microscope slides in Mowiol (Merck).

### Immunocytochemistry on whole mounts

Some preparations were treated as whole mounts, but the protocol for vibratome slices detailed above was applied. The only difference was a clearing step with 50% Glycerol in PBS for at least 1 h, followed by an incubation with 90% Glycerol in PBS for 1 h. Finally, whole tissue was mounted in 90% Glycerol in PBS containing 4% n-propyl-gallate (Sigma Aldrich) as an anti-fading agent. Several nerve cords underwent an enzyme treatment after the permeabilization step. Therefore, the tissue was washed in PBS at 35 °C for at least 15 min and incubated in 1 mg/ml Collagenase Dispase (Roche Diagnostics, Basel, Switzerland) dissolved in PBS for 35 min at 35 °C. Afterwards, preparations were washed three times in ice-cold PBS-TX 0.5%, followed by the blocking step (see above).

### Antibody characterization

The polyclonal antibody was raised against synthetic histamine, conjugated to succinylated keyhole limpet hemocyanine with carbodiimide. No cross-reaction with L-histidine or L-histidine containing peptides has been documented (ImmunoStar, data sheet cat. #22939). The same antibody has been used within several insect species, as well as a centipede and a tick [57, 70]. The total absence of immunolabeling in *Drosophila* null lines for histidine decarboxylase [63] indicates a high specificity of the antibody. In control experiments where the primary antibody was omitted, immunoreactive labeling was absent. Pre-adsorption with histamine eliminated labeling [69]. A monoclonal mouse anti-*Drosophila* synapsin antibody (“SYNORF1”, Developmental Studies Hybridoma Bank) raised against a *Drosophila* GST-synapsin fusion protein was applied. This antibody reacts with a highly conserved epitope, as it labels neuropil structures over a wide range of euarthropod taxa (e.g., [24, 75–77]). Additionally, in western blots of brain tissues of *Drosophila* and the crustacean *Coenobita clypeatus* identical bands were stained by the synapsin antibody, which suggests that the epitope for SYNORF1 is strongly conserved between *Drosophila* and *Coenobita* [76]. We used this antibody as a structural marker, facilitating orientation within the tissue of such diverse euarthropod species, especially within the fused nervous systems of Chelicerata.

### Image acquisition

Sections were examined with a Leica TSC SP5 II confocal microscope (cLSM). Z-series were processed with NIH ImageJ, v. 1.8 (Rasband WS, ImageJ, U.S. National Institutes of Health, Bethesda, MD, http://rsb.info.nih.gov/ij/), producing maximum projections. Images were processed in Adobe Photoshop 6.0 (San Jose, CA) using global contrast and brightness adjustment features as well as black and white inversion.

## Results

### *Thermobia domestica* (Zygentoma, Hexapoda)

**Fig. 1:**
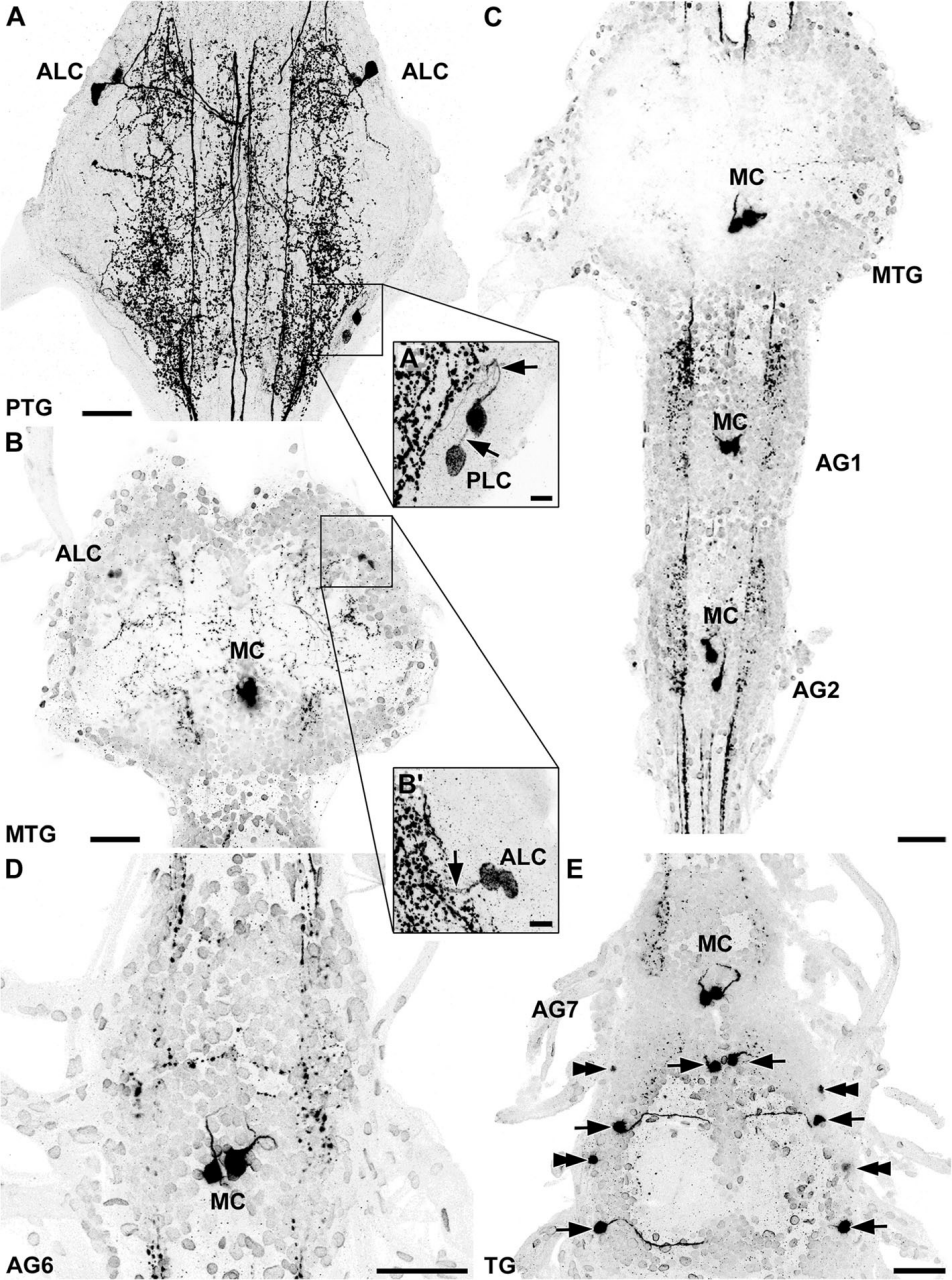
Distribution of histaminergic cells in the ventral nerve cord of *Thermobia domestica*. Maximum projections of cLSM stacks (ventral view, anterior to the top). **a** The prothoracic ganglion (PTG) contained up to three anterolateral (ALC) and two posterolateral cell bodies (PLC). **a’** The PLC showed a short anterior projection pattern (black arrows). **b** Additionally to the ALC, a pair of medial cell bodies (MC) could be identified in the metathoracic ganglion (MTG). **b’** The ALC projected in medial direction (black arrow), although the neurites could only be traced for a short distance. **c, d** Every ganglion except for the PTG possessed a pair of MC. **e** In the terminal ganglion (TG), three pairs of consistently labeled neurons (black arrows) and two additional faintly labeled somata groups (black double arrowheads) could be identified. Abbreviations: AG1–7: abdominal ganglia 1–7; ALC: anterolateral cell bodies; MC: medial cell bodies; MTG: metathoracic ganglion; PLC: posterolateral cell bodies; PTG: prothoracic ganglion; TG: terminal ganglion. Scale bars: a–e: 50 μm; a’, b’: 10 μm

The ventral nerve cord in *Thermobia domestica* is built up from three thoracic ganglia, seven abdominal ganglia, and the terminal ganglion, interconnected by longitudinal connectives [see also 25]. These connectives were condensed between the metathoracic, first, and second abdominal ganglia, as well as between the seventh abdominal ganglion and the terminal ganglion [25]. The hemineuromeres of each segmental ganglion were fused at the midline (Fig. 1). All histamine-immunoreactive somata were located in the ventral soma cortex.

**Fig. 2:**
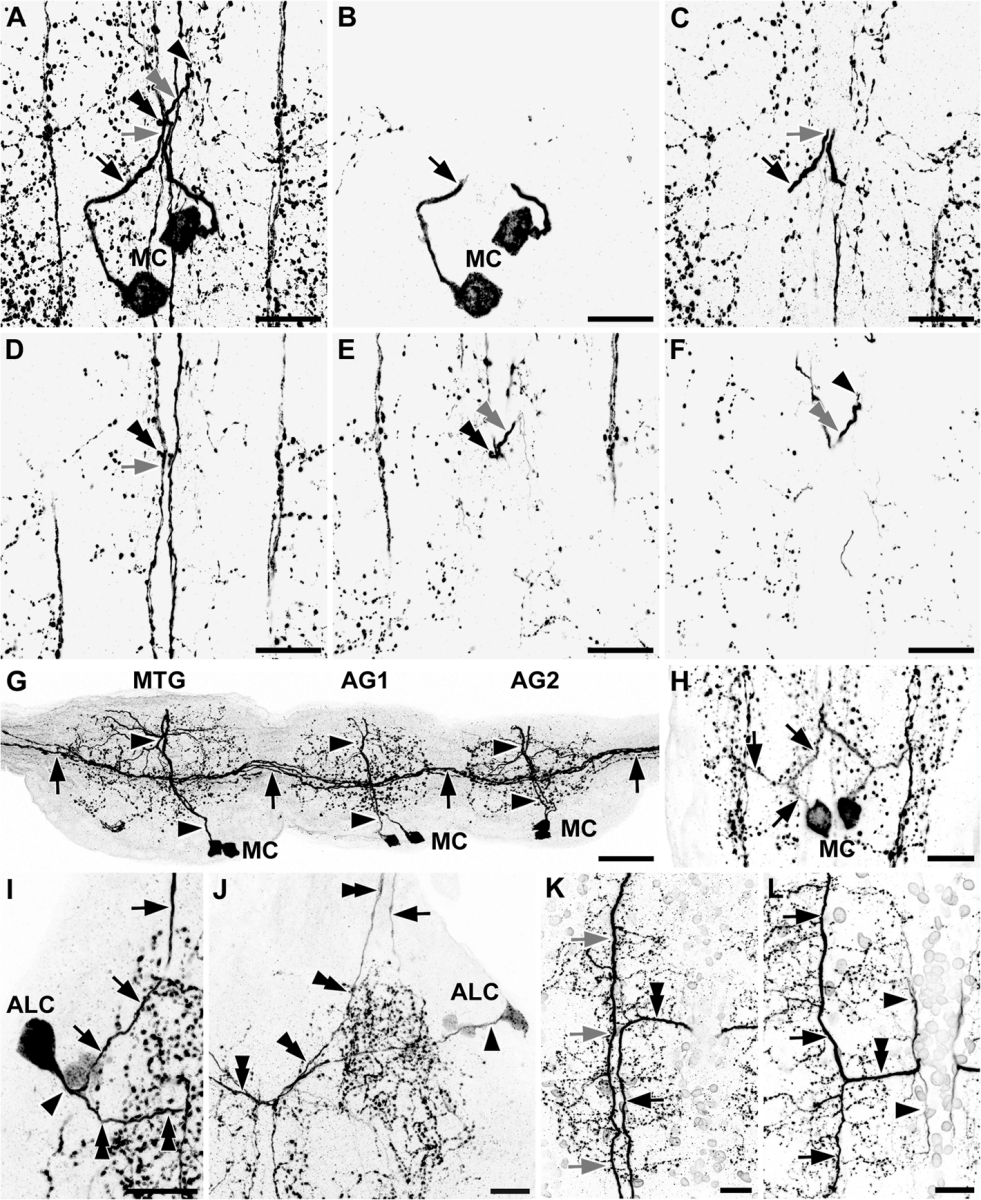
Morphology of primary neurites of the histaminergic neurons in *Thermobia domestica*. Maximum projections of cLSM stacks (a–f, h–l: ventral view, anterior to the top; g: sagittal view, anterior to the left, ventral to the bottom). **a–f** Ventral-to-dorsal series of maximum projections revealed a contralateral projection associated to medial cell bodies (MC). a shows the complete stack, which is split in b to f. Same arrow types point to the same positions throughout the series (e.g., grey arrows in a, c, d). **g** Sagittal view of the metathoracic (MTG), first and second abdominal ganglia (AG1–2), showing the dorsomedial projections of the MC (black arrowheads). A strongly labeled longitudinal tract consisting of several axons is also visible (black arrows). **h** Projections of the MC in the abdominal ganglia. The primary neurite splits shortly after leaving the cell body in an ipsilateral and a contralateral branch (black arrows). **i, j** The large anterolateral cell bodies in the prothoracic ganglion projected medially (black arrowheads). Shortly after leaving the cell body, the primary neurite split into an ipsilateral branch, which entered the anterior connective (black arrows). The other branch projected further medially, crossing the ganglionic midline (dashed line) and entered the contralateral anterior connective (black double arrowheads). **k, l** One longitudinal tract projects without any specific branching through the ganglia (grey arrows in k). A second tract (black arrows in k and l), showed a medial horizontal branch (black double arrowhead in l) toward the ganglionic midline (dashed line). Ipsilaterally to the midline, this horizontal branch split again in a faintly labeled anterior and posterior tract (black arrowheads in l). Abbreviations: AG1–2: abdominal ganglia 1–2; ALC: anterolateral cell bodies; MC: medial cell bodies; MTG: metathoracic ganglion. Scale bars: a-f, i-l: 25 μm; g: 50 μm; h: 20 μm

**Fig. 3:**
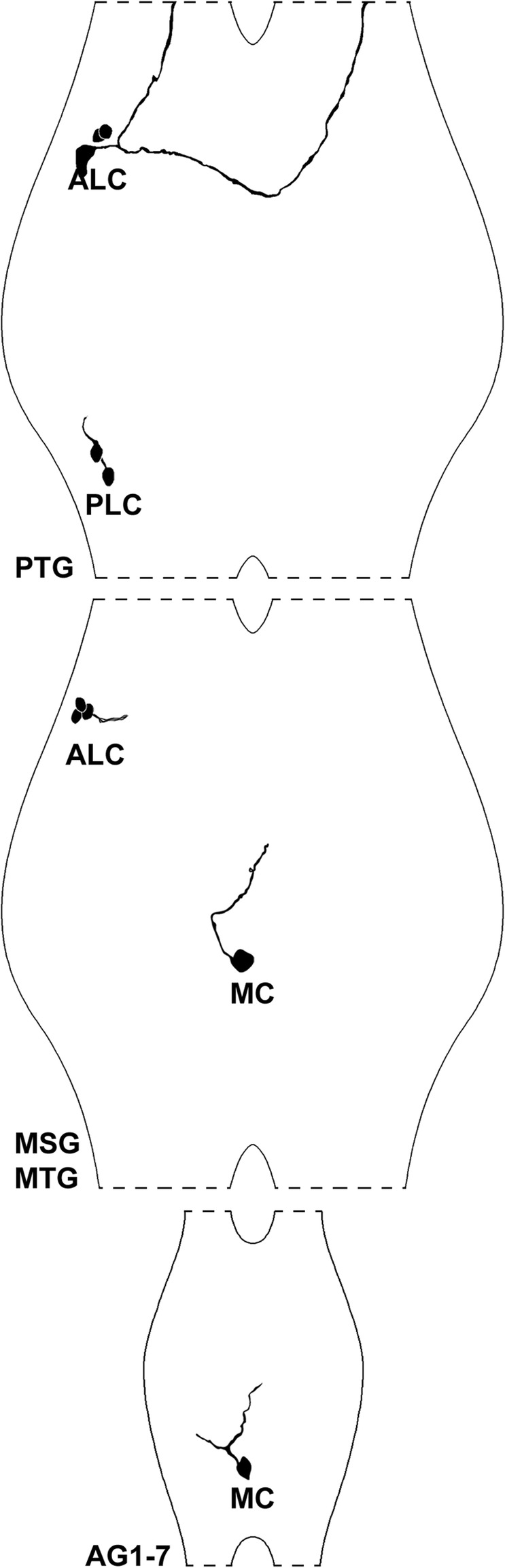
Reconstruction drawings of selected histaminergic neurons in the thoracic and abdominal ganglia of *Thermobia domestica*. Only neurons from the left hemiganglion were depicted for clarity. Abbreviations: AG1–7: abdominal ganglia 1–7; ALC: anterolateral cell bodies; MC: medial cell bodies; MSG: mesothoracic ganglion; MTG: metathoracic ganglion; PLC: posterolateral cell bodies; PTG: prothoracic ganglion

In all three thoracic ganglia, anterolateral immunoreactive neurons could be identified (Fig. 1a, b). However, number and intensity varied. One larger bilateral symmetric pair was detected consistently within the prothoracic ganglion (Fig. 1a), showing a conspicuous projection pattern. The primary neurite split into two branches (Figs. 2i, j; 3). The first branch turned anteriorly and projected into the ipsilateral connective (Figs. 2j; 3), while the second branch projected medially and crossed the midline. The branch in the contralateral hemiganglion turned anteriorly and entered the contralateral connective (Figs. 2i; 3). In some specimens, especially in sectioned preparations, one or two additional anterolateral pairs were visible in the prothoracic ganglion (Figs. 1i; 2i, j; 3). Furthermore, up to three anterolateral pairs were identified in the mesothoracic and metathoracic ganglia (Fig. 1b). The projections of these cells could be traced only for short distances in medial direction (Figs. 1b’; 3). Eight of 12 whole-mount preparations showed one or two faintly labeled anterolateral pairs, which sometimes occurred in only one hemiganglion. These neurons were reliably labeled bilaterally in the sectioned preparations.

Another immunoreactive cell group consisting of one or two bilateral symmetric pairs could be identified in posterolateral position in the prothoracic ganglion (Fig. 1a). Projections of these neurons could only be traced for a short distance in anterior direction (Figs. 1a’; 3). Immunoreactive neurons in a similar position were not found in the mesothoracic and metathoracic ganglion.

All preparations showed a bilateral symmetric pair of medial neurons in the mesothoracic and metathoracic ganglion (Fig. 1b, c) sending a primary neurite dorsomedially to the contralateral hemiganglion, where they joined a strongly immunoreactive longitudinal tract in the center of the hemiganglia (Figs. 2a-g; 3).

Each of the seven unfused abdominal ganglia contained a pair of bilateral symmetric, strongly immunoreactive neurons with somata positioned medially close to the midline (Fig. 1c-e). Their primary neurite split into two branches shortly after leaving the cell body (Figs. 2h; 3). One stayed on the ipsilateral side, whereas the other branch projected contralaterally, similar to the neurites of the medial neurons in the thorax (see above) (Figs. 2h; 3).

Most histamine-immunoreactive neurons were identified in the fused terminal ganglion. Three large pairs, located anteromedially, centrally, and posterolaterally, were intensely labeled in all preparations (Fig. 1e). The central and posterolateral soma possessed medially projecting neurites (Fig. 1e). Near the midline, these neurites bent anteriorly, but could not be followed further. Up to two smaller anterolateral and up to three posterolateral pairs were detected inconsistently within the terminal ganglion (Fig. 1e). No projections associated to these somata could be identified.

A prominent longitudinal tract consisting of at least two histamine immunoreactive axons was observed throughout the entire ventral nerve cord (Fig. 2g, k, l). One axon passed straight through the ganglion (Fig. 2k). A second longitudinal axon showed an additional horizontal medial projection near the center of every ganglion (Fig. 2k, l). Near the midline, this horizontal projection split again into an anteriorly and a posteriorly projecting branch, forming a H-like structure (Fig. 2l). The medial longitudinal branch of this “H” was only weakly labeled and appeared to be connected to the contralateral projection of the medial cells.

### *Xibalbanus tulumensis* (Remipedia, Crustacea)

**Fig. 4:**
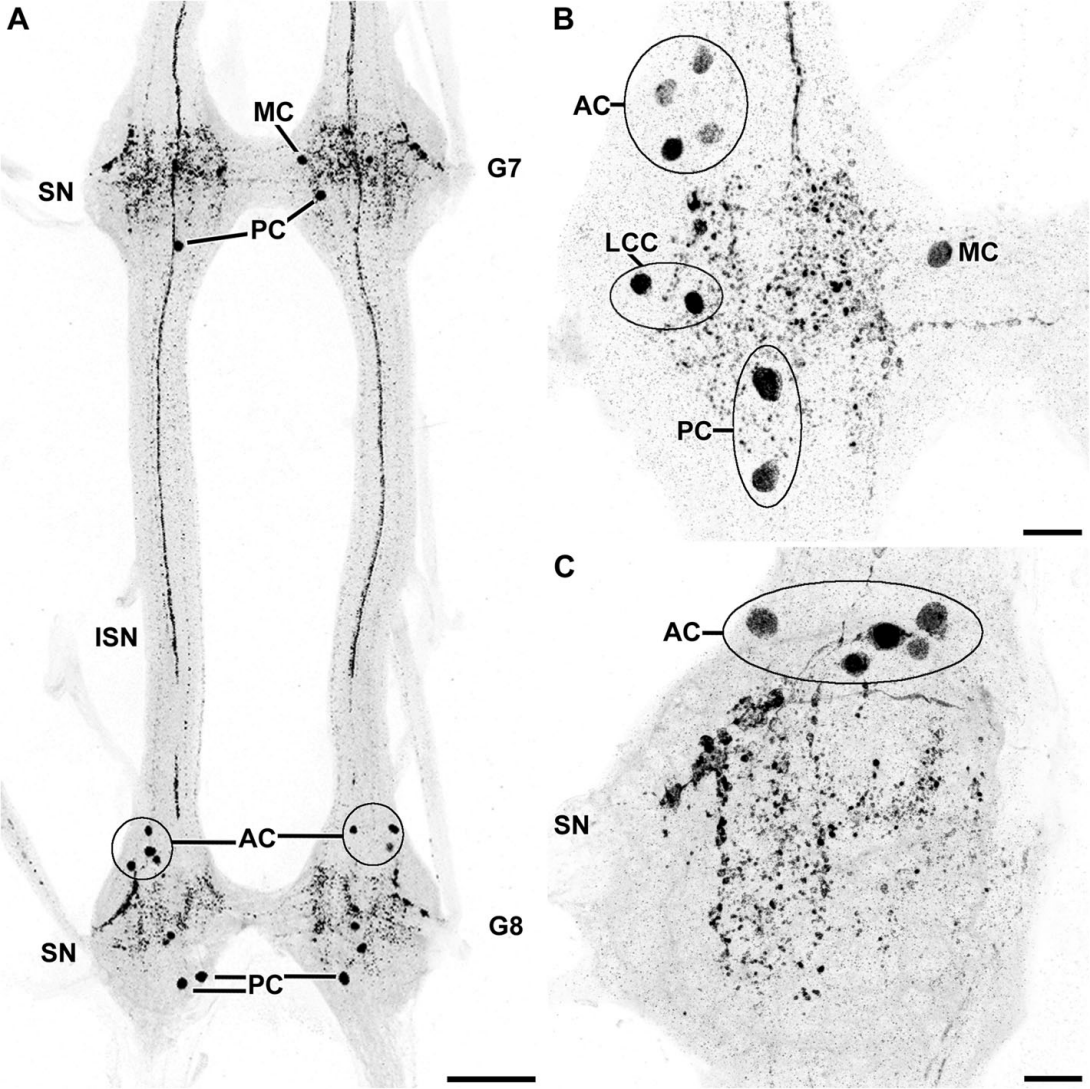
Histaminergic neurons in the ventral nerve cord of *Xibalbanus tulumensis*. Maximum projections of cLSM stacks (ventral view, anterior to the top). **a** Overview of the ganglia 7 and 8 (G7–8), showing the typical rope-ladder like arrangement and the variability concerning number and distribution of the histaminergic cell bodies. **b** Close-up of a representative hemiganglion, depicting anterior (AC), laterocentral (LCC), medial (MC) and posterior cell bodies (PC). **c** The cluster of anterior AC contained up to five immunoreactive neurons. Abbreviations: AC: anterior cell bodies; G7–8: ganglion 7–8; ISN: intersegmental nerve; LCC: laterocentral cell bodies; MC: medial cell bodies; PC: posterior cell bodies; SN: segmental nerve. Scale bars: a: 100 μm; b, c: 25 μm

The ventral nerve cord in the trunk segments of *Xibalbanus tulumensis* displayed a rope-ladder-like arrangement of separate metameric ganglia, which were interconnected by bilateral symmetric longitudinal connectives (Fig. 4a [24];). In adult *Xibalbanus tulumensis*, the number of trunk segments varies in an age-dependent manner [78]. Specimens used in this study possessed between 37 and 41 trunk segments/ganglia. The neuropils of the two bilateral segmental hemiganglia were connected by commissural fibers. Each ganglion was associated to an anterior and a posterior segmental nerve (Fig. 4a [24];). Additionally, two neurite bundles left each connective, with both of them eventually fusing, forming an intersegmental nerve (Fig. 4a [24];). All histamine-immunoreactive cell bodies were situated ventrally.

**Fig. 5:**
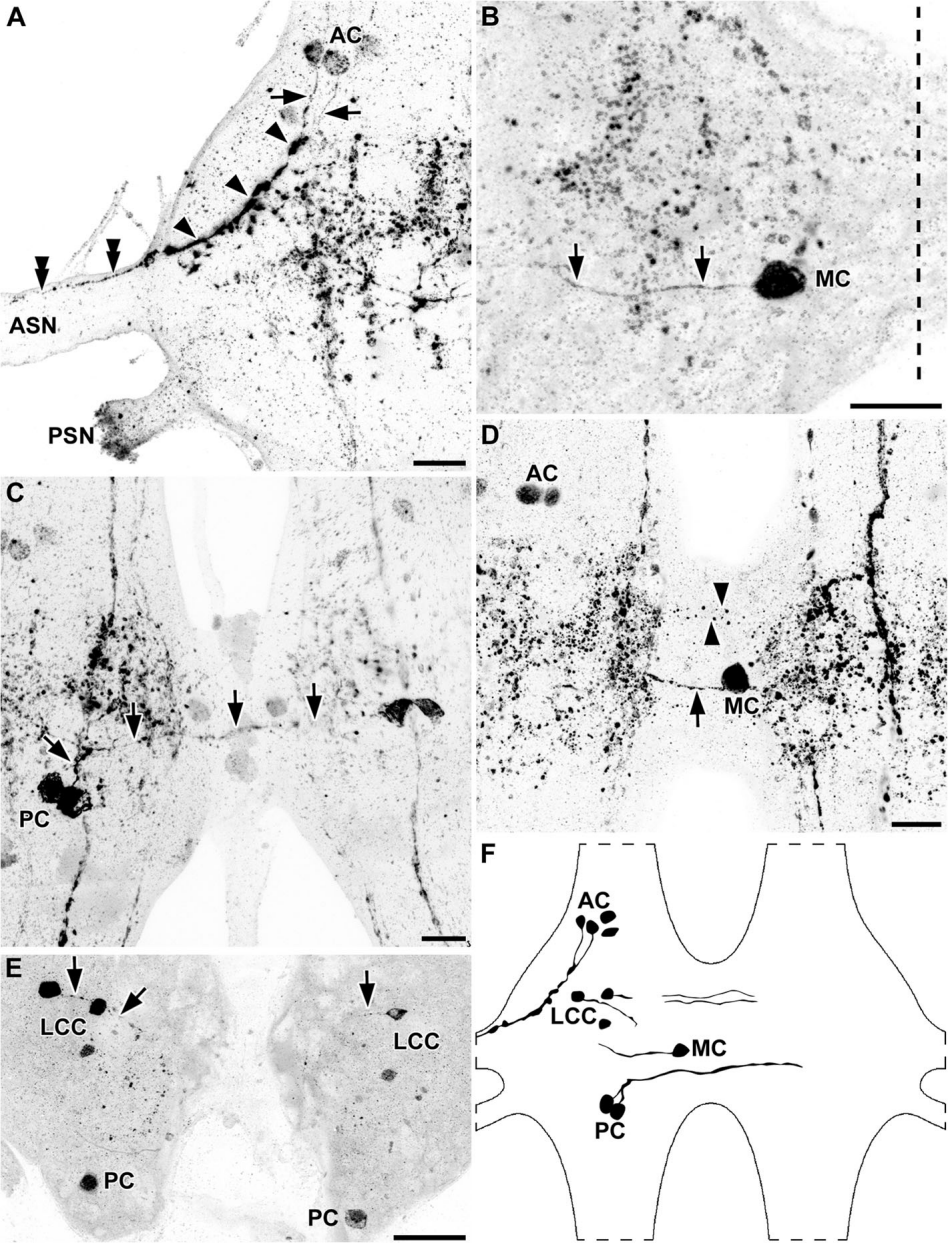
Morphology of primary neurites of histaminergic neurons in the ventral nerve cord of *Xibalbanus tulumensis*. Maximum projections of cLSM stacks (ventral view, anterior to the top). **a** At least two somata of the cluster of anterior cell bodies (AC) sent neurites (black arrows) into an elongated granular-structured region (black arrowheads), which is associated to the ipsilateral anterior segmental nerve (ASN) (black double arrowheads). **b** The medial cell bodies (MC) projected laterally toward the center of the ipsilateral hemiganglion (ganglionic midline indicated by dashed line). **c** The primary neurites of the posterior cell bodies (PC) projected anteromedially and crossed the midline. These neurites could be traced to the center of the contralateral hemiganglion until the signal was lost. **d** Besides the rather strongly immunoreactive posterior commissure (black arrow) originating in the PC, two faintly labeled contralateral axons could be identified anteriorly (black arrowheads). **e** Short medial projections associated to the laterocentral cell bodies could be identified (black arrows). **f** Reconstruction drawings of selected histaminergic neurons in a ganglion of the ventral nerve cord. Abbreviations: AC: anterior cell bodies; ASN: anterior segmental nerve; LCC: laterocentral cell bodies; MC: medial cell bodies; PC: posterior cell bodies; PSN: posterior segmental nerve. Scale bars: a–e: 25 μm

We identified four different groups of immunoreactive cell bodies based on their position within the ganglia (Figs. 4; 5). All of these groups were rather variable in terms of exact position and number of neurons. They could often only be assigned to a specific cluster by their characteristic neurite morphology. We observed 2–5 immunoreactive somata in an anterior position of each hemiganglion (Figs. 4a-c; 5a, d). The cell bodies were positioned directly posterior to the anterior connective and occurred over the entire width of the hemiganglion. At least two of these neurons projected their neurites posterolaterally into an elongated granular-structured region toward the anterior segmental nerve (Fig. 5a, f). From this conspicuous structure, some histaminergic neurons projected into the anterior nerve. It remains unclear, whether these neurons were connected to the anterior histaminergic somata, or if they arose in the elongated granular-structured area.

Furthermore, we reliably detected a medial immunoreactive cell body in each hemiganglion, located near the ganglionic midline (Figs. 4a, b; 5b, d). A clearly ipsilateral projection was present near the center of the hemiganglion (Fig. 5b, f).

At the posterior margin of each hemiganglion, two cell bodies were situated with a similar variability as the anterior cell group (Figs. 4a, b; 5c, e). Both cells projected their primary neurites anteromedially and contralaterally, crossing the midline at a posterior level (Fig. 5c, f). Additionally, two more faintly labeled neurites crossed the midline, forming an anterior histaminergic commissure (Fig. 5d, f). The origin of the later fibers could not be identified. However, the last group of immunoreactive cells—one to three somata in a laterocentral position (Figs. 4b; 5e)—sent short processes medially in direct orientation toward the anterior commissure, before the signal was lost (Fig. 5e, f).

### *Glomeris marginata* (Diplopoda, Myriapoda)

**Fig. 6:**
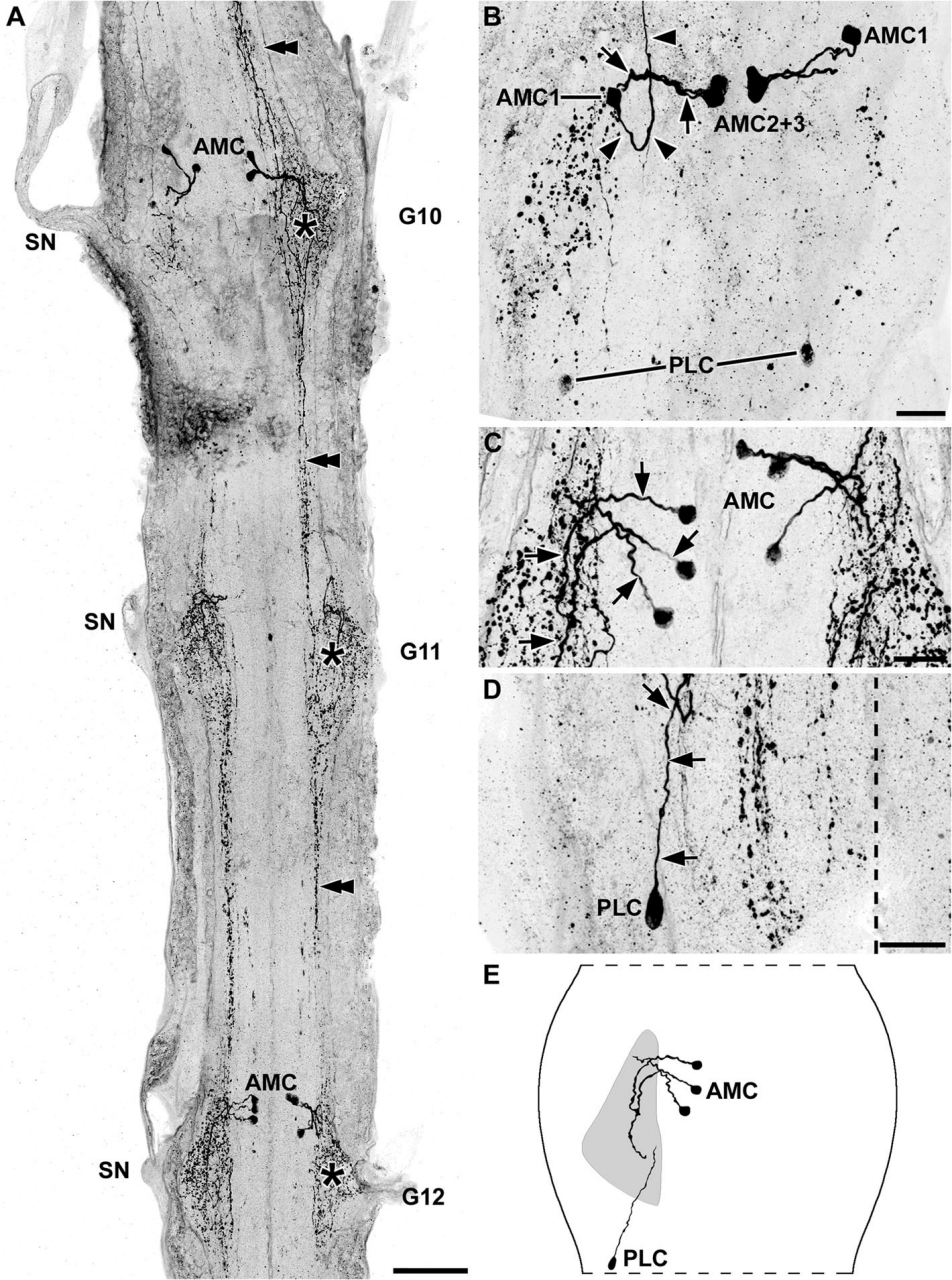
Distribution and projection pattern of histaminergic neurons in the ventral nerve cord of *Glomeris marginata*. Maximum projections of cLSM stacks (ventral view, anterior to the top). **a** Overview of the ganglia 10–12 (G10–12), illustrating the rather homogenous structure of the ventral nerve cord. The ganglia could be distinguished from the connectives by the segmental nerves (SN). The ganglia contained the anteromedial cell bodies (AMC) as well as a conspicuous triangular neuropil, revealed by dense histamine-immunoreactivity (asterisks). A strongly labeled longitudinal tract could be observed throughout the ventral nerve cord (double arrowhead). **b, c** Besides the AMC, each ganglion contained a posterolateral soma pair (PLC). The three AMC can be subdivided in a single cell (AMC1) and two closely associated cells (AMC2 + 3). All AMCs projected laterally and posteriorly into the triangular neuropilar structure (black arrows in b and c). In rare cases, the AMC1 performed a loop, growing first posteriorly, but then looping back anteriorly before its signal got lost (black arrowheads in b). **d** The PLC projected anterolaterally toward the center of the ipsilateral hemiganglion (black arrows; ganglionic midline indicated by dashed line). **e** Reconstruction drawings of selected histaminergic neurons in a ganglion of the ventral nerve cord. The triangular histamine-immunoreactive neuropil is highlighted in grey. Abbreviations: AMC1–3: anteromedial cell bodies 1–3; G10–12: ganglion 10–12; PLC: posterolateral cell bodies; SN: segmental nerve. Scale bars: a: 100 μm; b-d: 25 μm

As a typical representative of Diplopoda, *Glomeris marginata* possesses diplosegments. Thus, the number of tergites (12) does not match with the number of legs (17 in females, 19 in males) [79]. Whether or not the segmental fusion is also represented in the ganglia associated with the one diplosegment is not clearly evaluated in the literature (see [80] for further information). However, in *Glomeris marginata*, each pair of legs was associated with a separated ganglion, which could be identified as small swellings with a prominent segmental nerve root (Fig. 6a). Thus, males possessed 19 and females 17 ganglia, and no ganglionic fusion was observed within the diplosegments. The bilateral hemiganglia were strongly fused at the midline, without clear separation in an anterior and posterior commissure (Fig. 6a).

In general, histamine immunoreactivity was observed throughout the ventral nerve cord. Each ganglion showed strong varicose labeling of fibers in the hemineuromeres (Fig. 6). However, almost no immunoreactivity was observed near the midline and no contralateral projecting fibers identified (Fig. 6). This distribution of immunoreactive neuropilar regions could be recognized as an approximately triangular region in each hemiganglion (Fig. 6e).

In each ganglion, three consistently labeled cell somata could be detected anteromedially (Fig. 6a-c, e). Two of these cell somata were often clustering together, while the remaining cell was situated slightly apart, in a more posterior and/or lateral position (Fig. 6b). In most cases, all three neurons projected laterally before bending posteriorly toward the center of the ipsilateral neuromere (Fig. 6c, e). Occasionally, the projection of the single cell performed a loop, growing first posteriorly in the same direction as the other two axons, then looping back anteriorly before its signal was lost (Fig. 6b).

Furthermore, an additional immunoreactive soma was identified in posterolateral position (Fig. 6b, d, e). This cell body was labeled more faintly in comparison to the anteromedial cells and could not always be detected. The neurites associated to the posterolateral cell projected anteromedially toward the center of the ipsilateral hemiganglion (Fig. 6d, e). All immunoreactive somata were located ventrally.

### Chelicerata

**Fig. 7:**
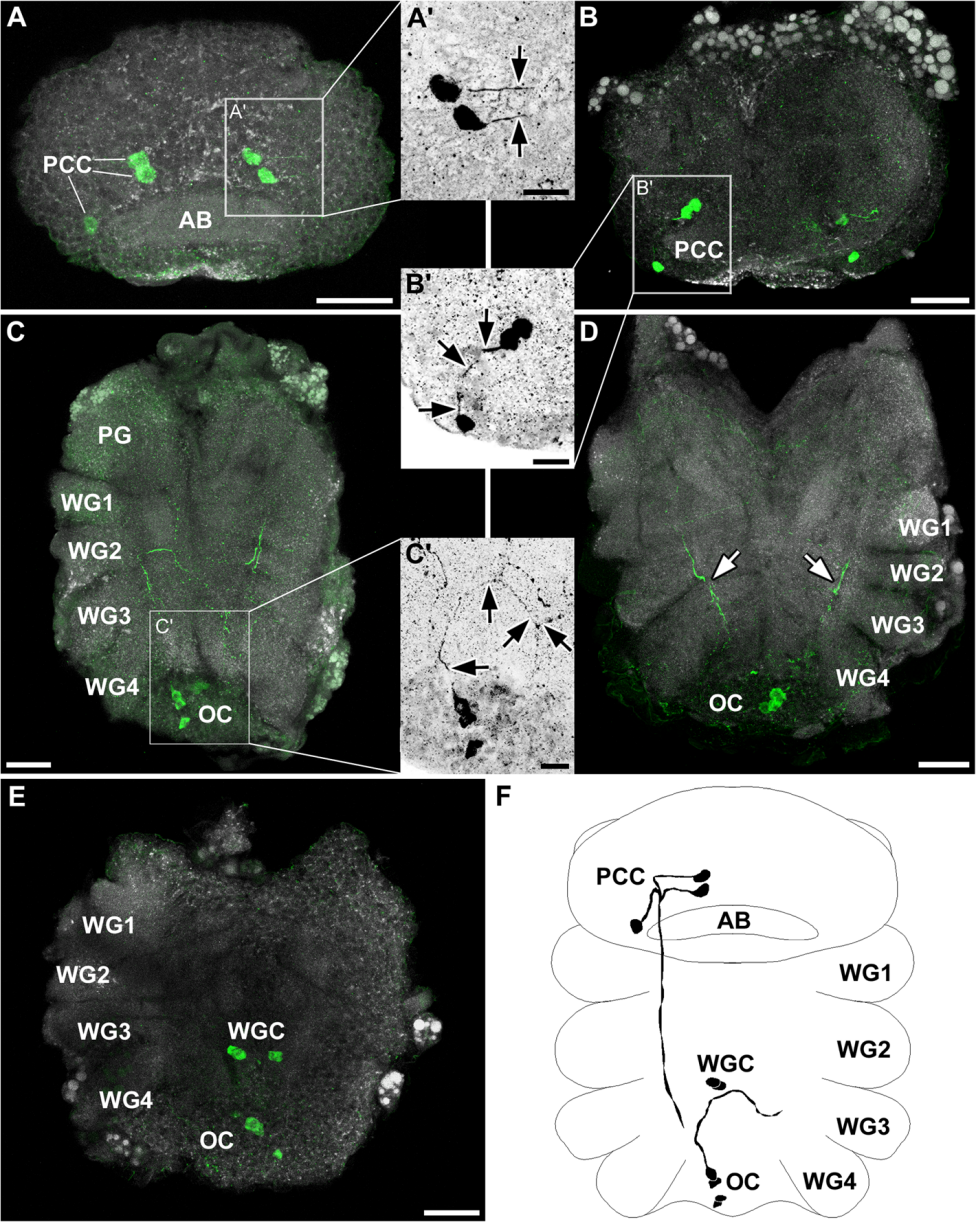
Histaminergic neurons in the central nervous system of *Chelifer cancroides*. Maximum projections of cLSM stacks (ventral view, anterior to the top), showing immunoreactivity of histamine (green) and synapsin (grey). **a, b** Three protocerebral histaminergic neurons (PCC) per hemiganglion were identified in the protocerebrum: two PCC were positioned anterodorsally to the arcuate body (AB), one laterally to the AB. **a’, b’** All PCC projected single neurites toward each other (black arrows), before turning posteroventrally. **c** Two pairs of immunoreactive cell bodies (OC) were identified in the fused opisthosomal ganglia. **c**’ A single contralateral projection arose from the OC (black arrows). **d** A conspicuous longitudinal immunoreactive tract projected throughout the length of the walking leg ganglia (white arrows). **e** A pair of histaminergic cell bodies (WGC) were found in the posterior regions of the prosoma, probably belonging to the walking leg ganglion 3. **f** Reconstruction drawings of selected histaminergic neurons in the central nervous system. Abbreviations: AB: arcuate body; OC: opisthosomal cell bodies; PCC: protocerebral cell bodies; PG: pedipalpal ganglion; WG1–4: walking leg ganglion 1–4; WGC: walking leg ganglion cell bodies. Scale bars: a–e: 50 μm; a’–c’: 25 μm

**Fig. 8:**
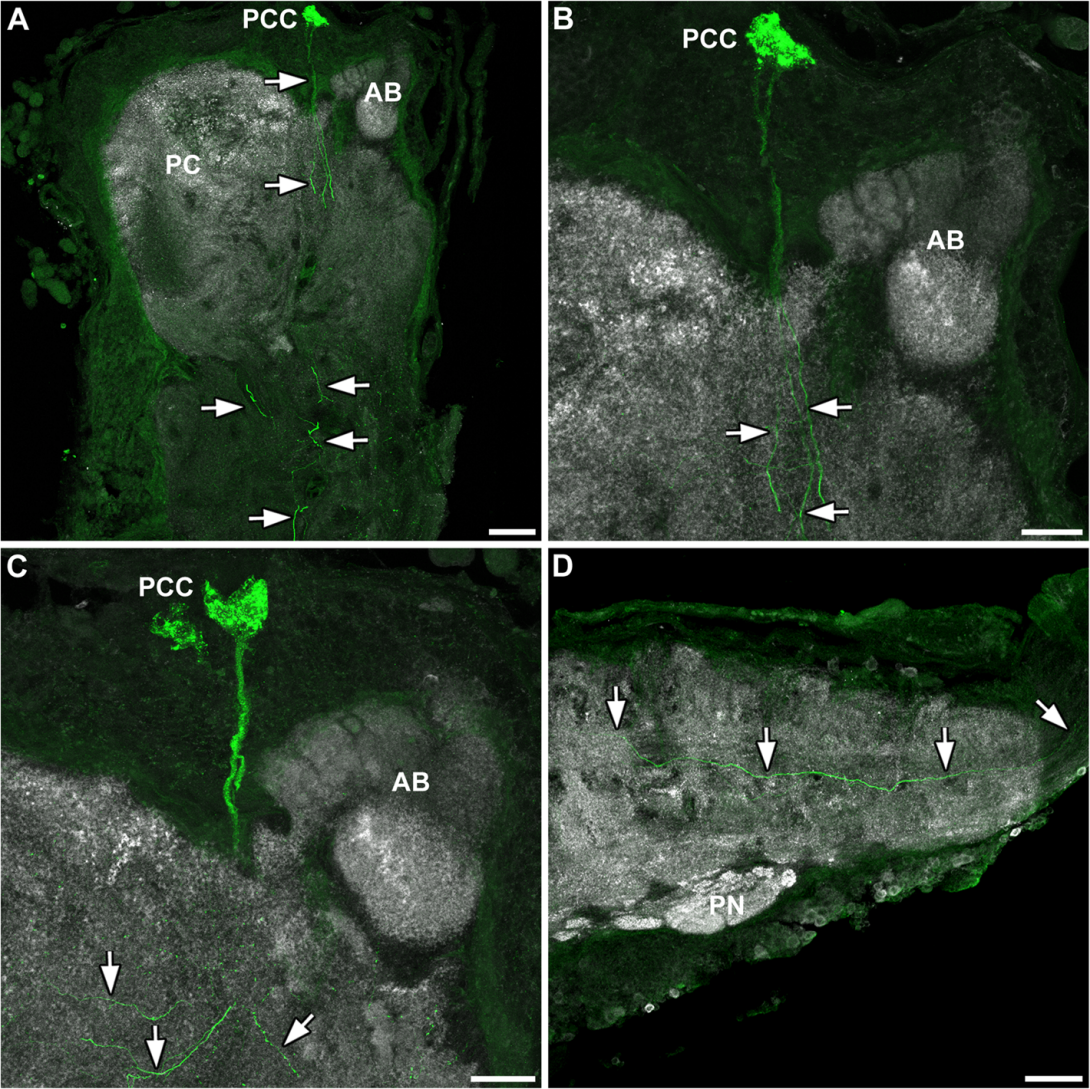
Histaminergic neurons in the central nervous system of *Euscorpius italicus*. Sagittal sections (anterior to the left, ventral to the bottom) showing immunoreactivity of histamine (green) and synapsin (grey). **a** Three protocerebral histaminergic neurons (PCC) were identified anterodorsally to the arcuate body (AB). These somata sent projections ventrally toward more caudal body regions (white arrows). **b** Close-up of the PCC in A, revealing a single primary neurite for each immunoreactive cell body (white arrows). **c** These neurites innervated the whole nervous system, projecting not only caudally, but also anteriorly and posteriorly (white arrowheads). **d** The primary neurites innervated the entire nervous system, including the most posterior regions of the mesosoma (white arrows). Abbreviations: AB: arcuate body; PC: protocerebrum; PCC; protocerebral cell bodies; PN: pectine neuropil. Scale bars: a, d: 100 μm; b, c: 50 μm

In general, the central nervous system of scorpions and pseudoscorpions shows a strong degree of fusion, complicating the identification of ganglionic structures [81, 82]. Therefore, we used an antibody against synaptic-rich regions as a structural marker for orientation (Figs. 7; 8). The nervous system is composed of the protocerebrum, deutocerebrum, tritocerebrum, the four walking leg neuropils, and the largely reduced opisthosomal ganglia [81, 82]. All these segmental neuromeres are fused to a so-called synganglion [81]. In scorpions, the synganglion is posteriorly connected to a nerve chain composed of three mesosomal and four metasomal ganglia [82].

#### *Chelifer cancroides* (Pseudoscorpiones)

In the pseudoscorpion *Chelifer cancroides*, we identified three consistently labeled pairs of histamine-immunoreactive somata positioned near the arcuate body in the syncerebrum. Two of these somata clustered together anteriorly to the arcuate body, whereas the third soma was located slightly posterolaterally and more ventrally (Fig. 7a, b, f). The pair projected their primary neurites laterally (Fig. 7a’, f), whereas the single soma sent its neurite in an anteromedial direction (Fig. 7b’, f). When reaching each other, the neurites extended ventrally and further posteriorly toward the fused leg ganglia (Fig. 7d, e).

In *Chelifer cancroides*, up to three histamine-immunoreactive groups, each consisting of two somata pairs, were labeled near the midline in the prosomal and opisthosomal ganglia. Two of these groups were situated in the fused opisthosomal ganglia (Fig. 7c-f). In one preparation, a single contralateral projection from one cell of the anterior opisthosomal pairs was detected (Fig. 7c’). Two pairs were identified in the posterior region of the prosomal walking leg ganglia (Fig. 7e-f); however a clear assignment of the neurons to a specific ganglion was not possible. Most likely, these neurons were associated with the third walking leg ganglion. No details concerning neurite morphology were observed for these immunoreactive neurons.

#### Euscorpius italicus (Scorpiones)

Three bilateral symmetric pairs of histamine-immunoreactive cells were found in the brain of *Euscorpius italicus* (Fig. 8). In contrast to the situation in pseudoscorpions, all three neurons were clustering closely together in an anterodorsal position to the arcuate body (Fig. 8a-c). Projections of these cells arborized ventrally and posteriorly throughout the entire synganglion, beginning in the protocerebrum, downwards to the deutocerebrum and to the upper posterior part of the tritocerebrum (Fig. 8). From there on, the neurites projected further posteriorly throughout the four walking leg ganglia (Fig. 8d), as observed in *Chelifer cancroides* (compare Figs. 7 and 8). These projections extended into the metasomal ganglia (not shown). Aside from the protocerebral somata, no immunoreactive cell bodies were identified in other regions of the central nervous system of *Euscorpius italicus*.

## Discussion

In the present report, we provide a detailed analysis of the histaminergic system in the ventral ganglia of representatives of each major euarthropod taxon. In the following discussion, we compare our newly generated data to those of previous investigations, presenting the first complete comparative account. Furthermore, we reconstruct ground patterns for those taxa, where histamine-immunoreactivity has been described and evaluate the phylogenetic value of this character set. Our interpretations, concerning the putative homology of neurons is, in addition to the shared neurotransmitter histamine, based on morphological criteria, namely somata positions and neurite morphologies [20].

### Histamine in the CNS of Chelicerata

**Fig. 9:**
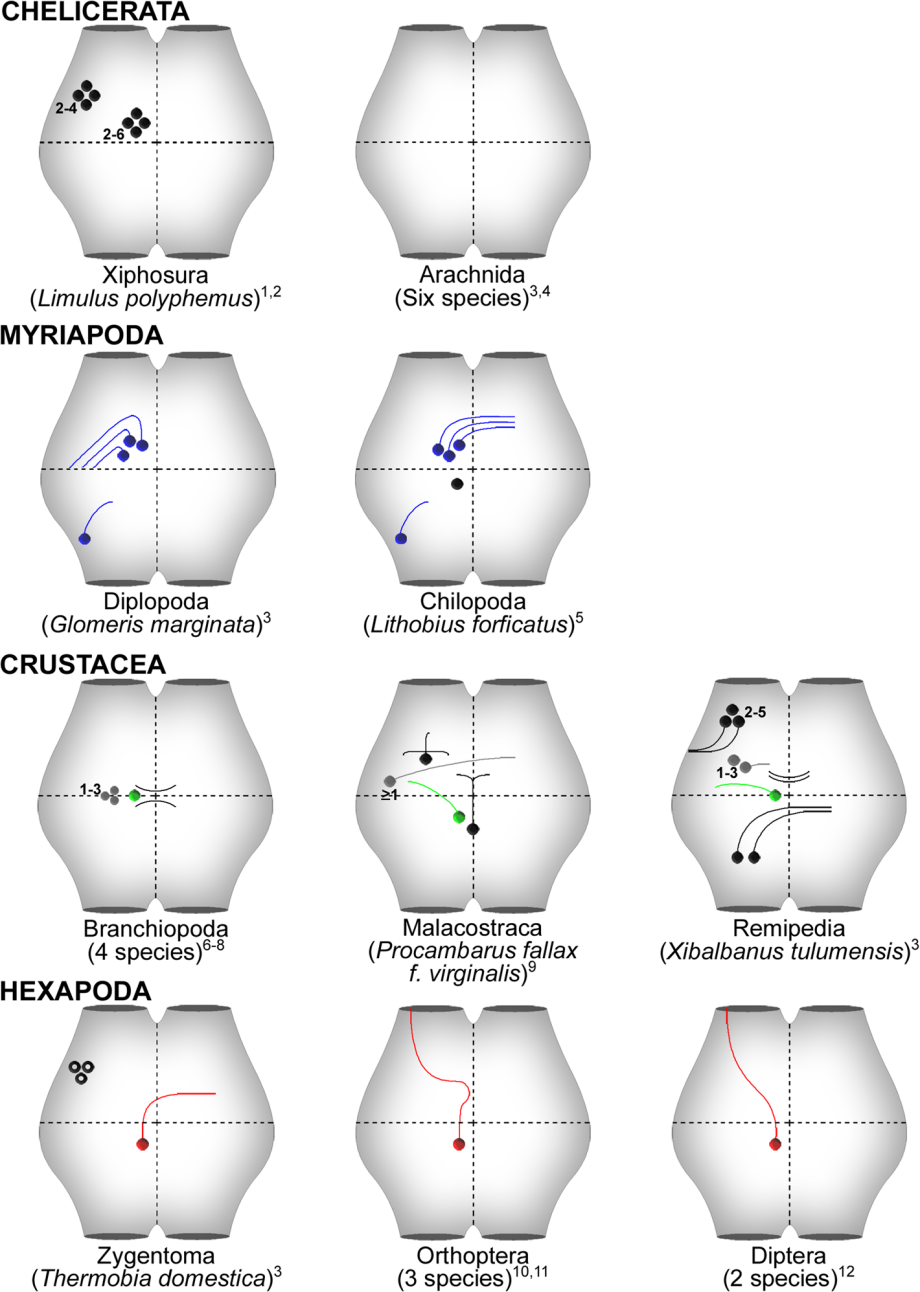
Patterns of histaminergic neurons in the ventral ganglia for the major euarthropod taxa. Blue, green, and red neurons are interpreted as homologous neurons within the respective taxa. Gray neurons in the crustacean taxa might be homologous, but this has to be proven in future studies. Black neurons do not have corresponding cells in other taxa. Numbers near cell groups depict the variability in cell number of the respective group. Open circles in the zygentoman pattern show neurons that are only found in the thoracic ganglia. Patterns are based on the original descriptions from 1: [42]; 2: [43]; 3: this study; 4: [45]; 5: [41]; 6: [47]; 7: [48]; 8: [49]; 9: [51]; 10: [52]; 11: [53]; 12: [55]

The histaminergic system in Chelicerata has been investigated in the horseshoe crab *Limulus polyphemus* (Xiphosura) [42, 43], several spiders [44, 45], the pseudoscorpion *Chelifer cancroides* as well as the scorpion *Euscorpius italicus* (this study) (Table 1). Below, we focus on histaminergic neurons within the central nervous system, but not on the distribution of histamine within chelicerate photoreceptors (e.g., [60]). The histaminergic transmitter system appears to be strongly conserved and restricted to three pairs of immunoreactive neurons situated in the brain in close vicinity to the arcuate body. Besides some immunoreactive neurons in the posterior region of the walking leg ganglia in the pseudoscorpion *Chelifer cancroides*, no immunoreactive somata have been observed in the ventral ganglia of Arachnida (Fig. 9). Only a single exception has been observed in the tick *Amblyomma americanum*, in which one histaminergic pair in the ventral ganglia extended neurites in most neuromeres of the synganglion [70]. In the xiphosuran *Limulus polyphemus,* clusters of immunoreactive neurons have been found in cheliceral, pedipalpal as well as prosomal ganglia (Fig. 9) [43]. Number and position of somata may vary, depending on the neuromere. No detailed information concerning projection pattern is available; however, a medial and a more lateral longitudinal fiber tract, as well as contralateral projections, can be observed in the original work by Harzsch and colleagues ([43]; see their Fig. 2). The authors did not discuss the situation in the opisthosomal ganglia; however, in their Fig. 2a, one or two laterally positioned somata can be identified in the first and second opisthosomal ganglia. Battelle et al. [42] mentioned a cluster of 15–20 cells in the cheliceral neuromere in adult specimens of *Limulus polyphemus*. As this study focused on the brain, no further information on other ventral neuromeres was provided. Nevertheless, this short statement may imply an increase in immunoreactive neurons per cluster during development also for the other neuromeres in *Limulus*.

Taking the current hypotheses of chelicerate phylogeny into account, the Xiphosura resemble one of the most basal taxa (reviewed in [14, 83], but see [15] for an alternative view on chelicerate phylogeny). Thus, clusters of histamine-immunoreactive neurons appear to represent the plesiomorphic condition and the loss of those neurons in arachnids must be interpreted as derived. A closer look at other chelicerate taxa will further refine this hypothesis. Examination of the pycnogonids, another rather basal clade within chelicerates, may reveal surprising results as already shown for serotonergic neurons [27]. These sea spiders are the only chelicerate taxon so far, where individually identifiable serotonergic neurons have been described, with some suggested homologies to mandibulate serotonergic neurons [27, 28].

### Myriapoda

The Myriapoda is the most underinvestigated group concerning the histaminergic system. Besides the description of histaminergic pathways in the optic ganglia of the chilopod *Scutigera coleoptrata* [61], this transmitter system focusing on the ventral nerve cord has only been investigated in another chilopod representative, *Lithobius forficatus* [41]. Comparing the histaminergic system of *Lithobius forficatus* [41] to that of *Glomeris marginata* (this study) reveals strong correspondences in somata distribution. Both species show three medially located immunoreactive pairs, which are slightly shifted toward the anterior half of the ganglion and are here interpreted as homologous neurons (blue neurons in Fig. 9). Nevertheless, the projection patterns of these anteromedial cells differ remarkably. Whereas the primary neurites project contralaterally in *Lithobius forficatus*, they stay on the ipsilateral side in *Glomeris marginata* (Fig. 9). Another homologous neuron with corresponding cell body position as well as neurite morphology was identified in both species in a posterolateral position (Fig. 9).

A single neuron has been described in *Lithobius forficatus* in a posteromedial position [41], but without a counterpart in *Glomeris marginata* (Fig. 9), presumably representing an apomorphy for this species.

### Crustacea

Within the Crustacea, several representatives covering different taxonomic groups have been investigated in the context of histaminergic neurons in the ventral ganglia (Table 1, Fig. 9). This includes three representatives of Malacostraca [50, 51], four species of Branchiopoda [47–49], as well as the remipede *Xibalbanus tulumensis* (this study). Furthermore, two species belonging to the Cirripedia are covered [46]. Compared to the other euarthropod lineages, the histaminergic system within Crustacea is far more diverse. The homologization of histaminergic neurons has been difficult, as some studies were undertaken with a different focus (e.g., the cephalic nervous system) or do not state relevant information, such as the number of cells in specific positions. Nevertheless, there are considerable data concerning the crustacean taxa Branchiopoda and Malacostraca, allowing the reconstruction of ground patterns.

#### Branchiopoda

In every species investigated, the histaminergic system within each ganglion contains two medially positioned, bilateral symmetric clusters with variable cell numbers. The two *Daphnia* species investigated by McCoole et al. [49] contained two to eight histaminergic cell bodies per segment, one pair being larger and more intensely labeled than the other cells. Although no information related to projection patterns of these neurons are given, a prominent longitudinal tract crossing the hemiganglia medially as well as a fine net of contralateral fibers connecting both hemiganglia is present ([49], see their Figs. 1 and 2). In *Artemia salina*, no exact cell numbers are available, but appear similar to those observed in the *Daphnia* species [47]. In *Triops cancriformis*, two to four medially situated somata occur within each ganglion, one pair being larger than the other cell bodies [48]. These neurons can be mono- or bipolar with some branches entering the anterior commissure projecting contralaterally, whereas other neurites stay ipsilateral [48].

Based on these descriptions, we suggest the following ground pattern for Branchiopoda. Each hemiganglion contains at least one larger histaminergic neuron, which may be accompanied by up to three smaller neurons (Fig. 9). As descriptions of projections were rather superficial and could not be clearly associated to certain neurons, our conclusion in this context remains hypothetical. Mono- or bipolar projections enter the longitudinal tracts and may be contra- or ipsilateral.

#### Malacostraca

Within the Malacostraca, three decapod species have been investigated. Mulloney and Hall [50] described the histaminergic system in *Homarus americanus* and *Pacifastacus leniusculus*, but did not mention any differences between the two species concerning cell body distribution. These authors proposed two pairs of serial homologous neurons in the ganglia of the ventral nerve cord. However, no information concerning projection patterns of these neurons was given. Rieger and Harzsch [51] investigated the development of histaminergic neurons in the ventral nerve cord of the marbled crayfish in detail and found a more complex situation than Mulloney and Hall [50]. Rieger and Harzsch [51] described four groups of serially repetitive neurons based on the position of the cell bodies: a lateral, an anterocentral, a medial and an unpaired midline group, all showing characteristic projection patterns (Fig. 9). The brightly labeled cell pair described by Mulloney and Hall [50] probably corresponds to the medial cell pair in the Marbled Crayfish, as already stated by Rieger and Harzsch [51]. The other cell pair mentioned by Mulloney and Hall [50] cannot be unambiguously assigned to histaminergic cells in the Marbled Crayfish. Due to the accuracy of the descriptions by Rieger and Harzsch [51], we adopted their results as the current pattern of histaminergic neurons in Malacostraca (Fig. 9).

#### Comparison of the histaminergic pattern of Branchiopoda, Malacostraca, and Remipedia

The reconstructed patterns of histaminergic neurons of these three crustacean taxa do not share many similarities at a first glance (Fig. 9). Based on a corresponding position and neurite morphology (if known), we suggest the medial neurons in Remipedia and Malacostraca, as well as the larger medial cell in Branchiopoda to be homologous (green neurons in Fig. 9). Further candidates for homologous neurons in Crustacea are the smaller group in Branchiopoda, and the anterocentral, as well as laterocentral, group in Malacostraca and Remipedia, respectively (grey neurons in Fig. 9). However, the exact projection pattern of these neurons remains unknown in Branchiopoda and Remipedia. Contralateral projections have been described in Branchiopoda, but have not clearly be assigned to specific neurons [47–49]. Similarly, we detected faintly contralateral immunoreactive fibers in the remipede *Xibalbanus tulumensis* (Fig. 5d). Whether these projections originate at the laterocentral cell bodies must be addressed in future studies. At least, a single lateral neuron projects contralaterally in the ground pattern of Malacostraca (Fig. 9). However, the number of lateral neurons is not clear. Rieger and Harzsch [51] described up to four neurons in embryonic stages of the marbled crayfish in lateral position, which were reduced in adults [51]. Thus, homology of these cells remains uncertain.

### Hexapoda

The hexapod histaminergic system has been described in three orthopteran [52–54] and two dipteran representatives [55], as well as a single zygentoman species (*Thermobia domestica*, this study). In all species, a single pair of medioventral histaminergic neurons has been described within every ganglion of the ventral nerve cord, except for the prothoracic ganglion. Due to the prominent similarities in position, these medial cells are interpreted as homologous neurons within Hexapoda (red neurons in Fig. 9). However, a conspicuous difference in the morphology of these medial neurons requires a closer look: Whereas the primary neurite in locusts and flies stayed consistently ipsilateral, we identified a primary contralateral projection in *Thermobia domestica*. We conclude that an evolutionary shift from a contralateral projection pattern in Zygentoma to a primary ipsilateral pathway occurred in locusts and flies. The investigation of phylogenetically intermediate hexapod taxa, such as mayflies and dragonflies, would be worthwhile in tracing the evolutionary transformation of this character.

We identified several additional inconsistently labeled immunoreactive neurons in the thoracic ganglia of *Thermobia domestica*, which have not been identified in other hexapod species. Anterolateral neurons were found in every thoracic ganglion, which indicates serial homology, at least concerning the thoracic ganglia (Fig. 9). Furthermore, we detected another set of neurons in posterolateral position restricted to the prothoracic ganglion, having no correspondences in other euarthropod species. In general, the pterygote species are totally devoid of immunoreactive somata in the prothoracic ganglion [52, 53, 55]. Notably, the investigation of the serotonin transmitter system in basal hexapod taxa such as Zygentoma and Archaeognatha also showed more immunoreactive neurons and led to the re-modeling of the ground pattern of serotonergic neurons for Hexapoda [25]. This may be a common phenomenon for Hexapoda, describing a more complex transmitter system in basal representatives, which have been simplified in winged insects. However, the additionally detected neurons and their projections in *Thermobia domestica* might also result from the remarkable progress in microscopic techniques as well as reflect the different focus pursued in the discussed studies.

### Evolution of the histaminergic transmitter system in Euarthropoda

When comparing the histaminergic systems of the euarthropod taxa, it is obvious that individually identifiable, serial homologous neurons in the ventral nerve cord are an apomorphy of the Mandibulata. In Arachnida, no serial repeated neurons have been identified so far (Fig. 9) and in the xiphosuran *Limulus polyphemus,* clusters with numbers of up to 20 immunoreactive neurons instead of single cells have been described for adult animals [42]. The lower numbers observed in larval stages demonstrate that the histaminergic system can undergo considerable changes in embryonal and larval development [51]. Thus, only adult specimens should be taken into account in the scope of the present comparative study. Interestingly, we identified several pairs of immunoreactive neurons in the prosomal and opisthosomal ganglia of the pseudoscorpion *Chelifer cancroides*. A similar observation has been made in a tick, in which a single histaminergic pair was identified in the prosomal ganglia [70]. However, these are not found in every ganglion of the ventral nerve cord, thus are not clearly serial homologous and are not incorporated into the ground pattern of this group (Fig. 9). The histaminergic neurons may have been reduced in certain neuromeres in *Chelifer cancroides*, e.g., due to miniaturization. Alternatively, this picture has to be interpreted as a link between the loss of repetitive neurons in spiders and a yet undiscovered set of serial homologous histaminergic neurons in an uninvestigated taxon of Chelicerata. Accordingly, a broader taxon sampling is highly desired. One promising candidate to start with would be the Pycnogonida. This taxon shows—in contrast to the serotonergic clusters in scorpions and xiphosurans [23]—individually identifiable serotonergic neurons in the ventral nerve cord [27]. An investigation of the histaminergic system might reveal further surprises associated to these fascinating animals.

Compared to the Chelicerata, the Mandibulata show single individually identifiable neurons in almost every ganglion of the ventral nerve cord, with often clearly traceable primary neurites. Histaminergic systems are rather conserved in Hexapoda and Myriapoda, and to a certain degree in Crustacea (Fig. 9). We identified several neurons, which are homologous within their respective major euarthropod taxon (four cells in Myriapoda, a single cell in Hexapoda, and at least one cell in Crustacea).

The current dataset suggests that the histaminergic transmitter system is rather well-conserved within the major taxa. It remains speculative whether some histaminergic neurons can be homologized across major taxa. Medial neurons in Crustacea and Hexapoda are the most promising candidates for homologization (green and red neurons in Fig. 9). In this context, Chapman et al. [70] suggested homology of the mesothoracic pair of histaminergic neurons based on the ascending projections toward mechanosensory centers in the brain throughout the euarthropods, but without discussing the aspect of serial homology. One of the medial neurons in Myriapoda might also be related to the medial cells in Tetraconata.

A major question in euarthropod phylogeny involves the relationship between crustaceans and hexapods. Remipedia are suggested to be the closest relative of Hexapoda (summarized in [71, 72]). Although this hypothesis has received more and more support from molecular sequence analyses, synapomorphies based on morphological comparisons are rather limited (but see [84]). In this context, no potentially homologous neurons between Remipedia and Hexapoda were identified in our study. Comparisons of serotonergic neurons in the ventral nerve cord of Tetraconata revealed a possible synapomorphy of Remipedia, Cephalocarida, and Hexapoda ([24, 25, 28]; but see [85] for a different interpretation).

## Conclusions

Our comprehensive review of the distribution and morphology of histaminergic interneurons in the ventral nerve cord throughout the Euarthropoda further supports the growing body of studies supporting the Mandibulata concept. Furthermore, we identified a set of neurons, which can be considered as homologous within the respective major taxon. However, these neurons show variability in their neurite morphology, which could be of interest for future studies on internal phylogeny of these taxa. Homologization of neurons between the four major euarthropod clades remains difficult.

In conclusion, the histaminergic system does contain useful information for our understanding of euarthropod phylogeny. This character set has considerable potential to resolve relationships within the major clades at a deeper level of taxonomy, but only a broader taxon sampling can support this assumption. Dacks et al. [57] described histamine-immunoreactive neurons in the antennal lobes of the insect order Hymenoptera, and showed extensive morphological modifications, which were in accordance with the evolutionary relationships in this group. In contrast, the serotonergic system seems more conserved at the level of euarthropods, as several serotonergic neurons have been suggested to be homologous within Chelicerata, Myriapoda, Crustacea, as well as Hexapoda. The integration of both character sets, the more conserved serotonergic system and the more variable histaminergic system, might further improve the resolution of neuroanatomical data matrices. The present contribution provides a comprehensive basis for such an ambitious endeavour.

## Data Availability

The data generated and/or analyzed during the current study are available from the corresponding author upon reasonable request.

## References

[1] Strausfeld NJ (2012). Arthropod brains. Evolution, functional elegance, and historical significance.

[2] Schmidt-Rhaesa A, Harzsch S, Purschke G (2016). Structure and evolution of invertebrate nervous systems.

[3] Holmgren N (1916). Zur vergleichenden Anatomie des Gehirns von Polychaeten, Onychophoren, Xiphosuren, Arachniden, Crustaceen, Myriopoden und Insekten. Kungliga Svenska Vetenskapsakademiens Handlingar.

[4] Hanström B (1928). Vergleichende Anatomie des Nervensystems der Wirbellosen Tiere unter Berücksichtigung seiner Funktion.

[5] Friedrich M, Tautz D (1995). Ribosomal DNA phylogeny of the major extant arthropod classes and the evolution of myriapods. Nature..

[6] Roeding F, Hagner-Holler S, Ruhberg H, Ebersberger I, von Haeseler A, Kube M, Reinhardt R, Burmester T (2007). EST sequencing of Onychophora and phylogenomic analysis of Metazoa. Mol Phylogenet Evol.

[7] Dunn CW, Hejnol A, Matus DQ, Pang K, Browne WE, Smith SA, Seaver E, Rouse GW, Obst M, Edgecombe GD, Sørensen MV, Haddock SH, Schmidt-Rhaesa A, Okusu A, Kristensen RM, Wheeler WC, Martindale MQ, Giribet G (2008). Broad phylogenomic sampling improves resolution of the animal tree of life. Nature..

[8] Giribet G, Edgecombe GD (2019). The phylogeny and evolutionary history of arthropods. Curr Biol.

[9] Rota-Stabelli O, Telford MJ (2008). A multi criterion approach for the selection of optimal outgroups in phylogeny: recovering some support for Mandibulata over Myriochelata using mitogenomics. Mol Phylogenet Evol.

[10] Wägele JW, Kück P, Wägele JW, Bartolomaeus J (2014). Arthropod phylogeny and the origin of Tracheata (= Atelocerata) from Remipedia-like ancestors. Deep metazoan phylogeny: the backbone of the tree of life.

[11] Jenner RA (2010). Higher-level crustacean phylogeny: consensus and conflicting hypotheses. Arthropod Struct Dev..

[12] Fernández R, Edgecombe GD, Giribet G (2016). Exploring phylogenetic relationships within Myriapoda and the effects of matrix composition and occupancy on Phylogenomic reconstruction. Syst Biol.

[13] Fernández R, Edgecombe GD, Giribet G (2018). Phylogenomics illuminates the backbone of the Myriapoda tree of life and reconciles morphological and molecular phylogenies. Sci Rep.

[14] Giribet G (2018). Current views on chelicerate phylogeny—a tribute to Peter Weygoldt. Zool Anz.

[15] Ballesteros Jesús A, Sharma Prashant P (2019). A Critical Appraisal of the Placement of Xiphosura (Chelicerata) with Account of Known Sources of Phylogenetic Error. Systematic Biology.

[16] Harzsch S (2006). Neurophylogeny: architecture of the nervous system and a fresh view on arthropod phylogeny. Integr Comp Biol.

[17] Harzsch S (2007). The architecture of the nervous system provides important characters for phylogenetic reconstructions: examples from the Arthropoda. Species, Phylogeny and Evolution.

[18] Strausfeld NJ, Andrew DR (2011). A new view of insect-crustacean relationships I. inferences from neural cladistics and comparative neuroanatomy. Arthropod Struct Dev..

[19] Wolff GH, Thoen HH, Marschall J, Sayre ME, Strausfeld NJ (2017). An insect-like mushroom body in a crustacean brain. eLIFE..

[20] Kutsch W, Breidbach O, Evans PD (1994). Homologous structures in the nervous system of Arthropoda. Adv. Insect Physiol. 24.

[21] Harzsch S, Waloszek D (2000). Serotonin-immunoreactive neurons in the ventral nerve cord of Crustacea: a character to study aspects of arthropod phylogeny. Arthropod Struct Dev..

[22] Harzsch S (2003). Evolution of identified arthropod neurons: the serotonergic system in relation to engrailed-expressing cells in the embryonic ventral nerve cord of the american lobster *Homarus americanus* Milne Edwards, 1873 (Malacostraca, Pleocyemata, Homarida). Dev Biol.

[23] Harzsch S (2004). Phylogenetic comparison of serotonin-immunoreactive neurons in representatives of the Chilopoda, Diplopoda, and Chelicerata: implications for arthropod relationships. J Morphol.

[24] Stemme T, Iliffe TM, von Reumont BM, Koenemann S, Harzsch S, Bicker G (2013). Serotonin-immunoreactive neurons in the ventral nerve cord of Remipedia (Crustacea): support for a sister group relationship of Remipedia and Hexapoda?. BMC Evol Biol.

[25] Stemme T, Stern M, Bicker G (2017). Serotonin-containing neurons in basal insects: in search of ground patterns among Tetraconata. J Comp Neurol.

[26] Stegner MEJ, Brenneis G, Richter S (2014). The ventral nerve cord in Cephalocarida (Crustacea): new insights into the ground pattern of Tetraconata. J Morphol.

[27] Brenneis G, Scholtz G (2015). Serotonin-immunoreactivity in the ventral nerve cord of Pycnogonida – support for individually identifiable neurons as ancestral feature of the arthropod nervous system. BMC Evol Biol.

[28] Sombke A, Stemme T (2017). Serotonergic neurons in the ventral nerve cord of Chilopoda - a mandibulate pattern of individually identifiable neurons. BMC Zool Lett.

[29] Watson AHD (1986). The distribution of GABA-Iike immunoreactivity in the thoracic nervous system of the locust *Schistocerca gregaria*. Cell Tissue Res.

[30] Wiens T, Wolf H (1993). The inhibitory motoneurons of crayfish thoracic limbs: identification, structures, and homology with insect common inhibitors. J Comp Neurol.

[31] Wolf H, Lang DM (1994). Origin and clonal relationship of common inhibitory motoneurons CI1 and CI3 in the locust CNS. J Neurobiol.

[32] Wolf H, Harzsch S (2002). Evolution of the arthropod neuromuscular system. 2. Inhibitory innervation of the walking legs of a scorpion: *Vaejovis spinigerus* (wood, 1863), Vaejovidae, Scorpiones, Arachnida. Arthropod Struct Dev..

[33] Harzsch S, Müller CHG, Wolf H (2005). From variable to constant cell numbers: cellular characteristics of the arthropod nervous system argue against a sister-group relationship of Chelicerata and “Myriapoda” but favour the Mandibulata concept. Dev Genes Evol.

[34] Duve H, Thorpe A (1994). Distribution and functional significance of Leu-callatostatins in the blowfly *Calliphora vomitoria*. Cell Tissue Res.

[35] Dircksen H, Skiebe P, Abel B, Agricola H, Buchner K, Muren JE, Nässel DR (1999). Structure, distribution, and biological activity of novel members of the allatostatin family in the crayfish *Orconectes limosus*. Peptides..

[36] Walker RJ, Papaioannou S, Holden-Dye L (2009). A review of FMRFamide- and RFamide-like peptides in Metazoa. Invertebr Neurosci.

[37] Christie AE, Nolan DH, Ohno P, Hartline N, Lenz PH (2011). Identification of chelicerate neuropeptides using bioinformatics of publicly accessible expressed sequence tags. Gen Comp Endocrinol.

[38] Jékely G (2013). Global view of the evolution and diversity of metazoan neuropeptide signaling. Proc Natl Acad Sci U S A.

[39] Christie AE (2015). Neuropeptide discovery in *Symphylella vulgaris* (Myriapoda, Symphyla): in silico prediction of the first myriapod peptidome. Gen Comp Endocrinol.

[40] Swales LS, Evans PD. Distribution of SchistoFLRFamide-like immunoreactivity in the adult ventral nervous system of the locust. *Schistocerca gregaria* Cell Tissue Res. 1995;281:339–48. 10.1007/BF00583402 7648627

[41] Schendel V, Kenning M, Sombke A (2018). A comparative analysis of the ventral nerve cord of *Lithobius forficatus* (Lithobiomorpha): morphology, neuroanatomy, and individually identifiable neurons. Arthropod Syst Phylogeny.

[42] Battelle BA, Calman BG, Hart MK (1999). Cellular distribution and functions of histamine, octopamine, and serotonin in the peripheral visual system, brain, and circumesophageal ring of the horseshoe crab *Limulus polyphemus*. Microsc Res Tech.

[43] Harzsch S, Wildt M, Battelle BA, Waloszek D (2005). Immunohistochemical localization of neurotransmitters in the nervous system of larval *Limulus polyphemus* (Chelicerata, Xiphosura): evidence for a conserved protocerebral architecture in Euarthropoda. Arthropod Struct Dev.

[44] Schmid A, Duncker M (1993). Histamine immunoreactivity in the central nervous system of the spider *Cupiennius salei*. Cell Tissue Res.

[45] Schmid A, Becherer C (1999). Distribution of histamine in the CNS of different spiders. Microsc Res Tech.

[46] Callaway JC, Stuart AE (1999). The distribution of histamine and serotonin in the barnacle’s nervous system. Microsc Res Tech.

[47] Harzsch S, Glötzner J (2002). An immunohistochemical study of structure and development of the nervous system in the brine shrimp *Artemia salina* Linnaeus, 1758 (Branchiopoda, Anostraca) with remarks on the evolution of the arthropod brain. Arthropod Struct Dev..

[48] Fritsch M, Richter S (2010). The formation of the nervous system during larval development in *Triops cancriformis* (Bosc) (Crustacea, Branchiopoda): an immunohistochemical survey. J Morphol.

[49] McCoole MD, Baer KN, Christie AE (2011). Histaminergic signaling in the central nervous system of *Daphnia* and a role for it in the control of phototactic behavior. J Exp Biol.

[50] Mulloney B, Hall W (1991). Neurons with histamine-like immunoreactivity in the segmental and stomatogastric nervous system of the crayfish *Pacifastacus lenisculus* and the lobster *Homarus americanus*. Cell Tissue Res.

[51] Rieger V, Harzsch S (2008). Embryonic development of the histaminergic system in the ventral nerve cord of the marbled crayfish (Marmorkrebs). Tissue Cell.

[52] Pätschke A, Bicker G (2011). Development of histamine-immunoreactivity in the central nervous system of the two locust species *Schistocerca gregaria* and *Locusta migratoria*. Microsc Res Tech.

[53] Hörner M, Helle J, Schürmann FW. The distribution of histamine-immunoreactive neurons in the ventral nerve cord of the cricket. *Gryllus bimaculatus* Cell Tissue Res. 1996;286:393–405. 10.1007/s004410050709 8929342

[54] Hörner M (1999). Cytoarchitecture of histamine-, dopamine-, serotonin- and octopamine-containing neurons in the cricket ventral nerve cord. Microsc Res Tech.

[55] Nässel DR, Pirvola U, Panula P (1990). Histaminelike immunoreactive neurons innervating putative neurohaemal areas and central neuropil in the thoraco-abdominal ganglia of the flies *Drosophila* and *Calliphora*. J Comp Neurol.

[56] Sachse S, Peele P, Silbering A, Gühmann M, Galizia C (2006). Role of histamine as a putative inhibitory transmitter in the honeybee antennal lobe. Front Zool.

[57] Dacks AM, Reisenman CE, Paulk AC, Nighorn AJ (2010). Histamine-immunoreactive local neurons in the antennal lobes of the Hymenoptera. J Comp Neurol.

[58] Loesel R, Homberg U (1999). Histamine-immunoreactive neurons in the brain of the cockroach *Leucophaea maderae*. Brain Res.

[59] Nässel DR (1999). Histamine in the brain of insects: a review. Microsc Res Tech.

[60] Battelle BA (2006). The eyes of *Limulus polyphemus* (Xiphosura, Chelicerata) and their afferent and efferent projections. Arthropod Struct Dev..

[61] Sombke A, Harzsch S (2015). Immunolocalization of histamine in the optic neuropils of Scutigera coleoptrata (Myriapoda: Chilopoda) reveals the basal organization of visual systems in Mandibulata. Neurosci Lett.

[62] Buchner E, Buchner S, Burg M, Hofbauer A, Pak W, Pollack I (1993). Histamine is a major mechanosensory neurotransmitter candidate in *Drosophila melanogaster*. Cell Tissue Res.

[63] Melzig J, Buchner S, Wiebel F, Wolf R, Burg M, Pak WL, Buchner E (1996). Genetic depletion of histamine from the nervous system of *Drosophila* eliminates specific visual and mechanosensory behavior. J Comp Physiol A.

[64] Fabian R, Seyfarth EA (1997). Acetylcholine and histamine are transmitter candidates in identifiable mechanosensitive neurons of the spider *Cupiennius salei*: an immunocytochemical study. Cell Tissue Res.

[65] Murphy BF, Larimer JL (1991). The effects of various neurotransmitters and some of their agonists and antagonists on the crayfish abdominal positioning system. Comp Biochem Physiol.

[66] Claiborne BJ, Selverston AI (1984). Histamine as a neurotransmitter in the stomatogastric nervous system of the spiny lobster. J Neurosci.

[67] Hashemzadeh-Gargari H, Freschi JE (1992). Histamine activates chloride conductance in motor neurons of the lobster cardiac ganglion. J Neurophysiol.

[68] Skiebe P, Corrette B, Wiese K (1990). Evidence that histamine is the inhibitory transmitter of the auditory interneuron ON1 of crickets. Neurosci Lett.

[69] Bradley SP, Chapman PD, Lizbinski KM, Daly KC, Dacks AM (2016). A flight sensory-motor to olfactory processing circuit in the moth *Manduca sexta*. Front Neural Circuits.

[70] Chapman PD, Bradley SP, Haught EJ, Riggs KA, Haffar MM, Daly KC, Dacks AM (2017). Co-option of a motor-to-sensory histaminergic circuit correlates with insect flight biomechanics. Proc R Soc B.

[71] Schwentner M, Combosch DJ, Nelson JP, Giribet G (2017). A phylogenomic solution to the origin of insects by resolving crustacean-hexapod relationships. Curr Biol.

[72] Lozano-Fernandez J, Giacomelli M, Fleming JF, Chen A, Vinther J, Thomsen PF, Glenner H, Palero F, Legg DA, Iliffe TM, Pisani D, Olesen J (2019). Pancrustacean evolution illuminated by taxon-rich genomic-scale data sets with an expanded remipede sampling. Genome Biol Evol.

[73] Hoenemann M, Neiber MT, Humphreys WF, Iliffe TM, Li D, Schram FR, Koenemann S (2013). Phylogenetic analysis and systematic revision of Remipedia (Nectiopoda) from Bayesian analysis of molecular data. J Crust Biol.

[74] Yager J (1987). *Speleonectes tulumensis*, n. sp. (Crustacea, Remipedia) from two anchialine cenotes of the Yucatan peninsula, Mexico. Stygologia.

[75] Fabian-Fine R, Volknandt W, Seyfarth EA (1999). Peripheral synapses at identifiable mechanosensory neurons in the spider *Cupiennius salei*: synapsin-like immunoreactivity. Cell Tissue Res.

[76] Harzsch S, Hansson BS (2008). Brain architecture in the terrestrial hermit crab *Coenobita clypeatus* (Anomura, Coenobitidae), a crustacean with a good aerial sense of smell. BMC Neurosci.

[77] Sombke A, Harzsch S, Hansson BS (2011). Organization of deutocerebral neuropils and olfactory behavior in the centipede *Scutigera coleoptrata* (Linnaeus, 1758) (Myriapoda: Chilopoda). Chem Senses.

[78] Koenemann S, Iliffe TM. Class Remipedia. In: von Vaupel Klein JC, Charmantier-Daures M, Schram FR, editors. Treatise on Zoology—Anatomy, Taxonomy, Biology. The Crustacea. Vol. 4 Part A. Leiden: Brill; 2013. p. 125–177.

[79] Janssen R, Prpic NM, Damen WGM (2006). A review of the correlation of tergites, sternites, and leg pairs in diplopods. Front Zool.

[80] Sombke Andy, Rosenberg Jörg (2015). Diplopoda — nervous and neuroendocrine systems. Treatise on Zoology - Anatomy, Taxonomy, Biology. The Myriapoda, Volume 2.

[81] Lehmann T, Melzer R, Hörnig MK, Michalik P, Sombke A, Harzsch S, Schmidt-Rhaesa A, Harzsch S, Purschke G (2016). Arachnida (excluding Scorpiones). Structure and evolution of invertebrate nervous systems.

[82] Wolf H, Schmidt-Rhaesa A, Harzsch S, Purschke G (2016). Scorpiones. Structure and evolution of invertebrate nervous systems.

[83] Dunlop JA (2010). Geological history and phylogeny of Chelicerata. Arthropod Struct Dev..

[84] Moura G, Christoffersen ML (1996). The system of the mandibulate arthropods: Tracheata and Remipedia as sister groups, “Crustacea” nonmonophyletic. J Comp Biol.

[85] Stegner MEJ, Brenneis G, Richter S (2014). The ventral nerve cord in Cephalocarida (Crustacea): new insights into the ground pattern of Tetraconata. J Morphol.

